# Heroes of peer review: Hyongbum (Henry) Kim

**DOI:** 10.1186/s13059-016-1067-0

**Published:** 2016-09-29

**Authors:** Hyongbum Kim

**Affiliations:** Department of Pharmacology and Brain Korea 21 PLUS Project for Medical Science, Yonsei University College of Medicine, Seoul, 03722 South Korea

## Abstract

Peer reviewers are the unsung heroes of science. We celebrate reviewers through a series of interviews with people who have made particularly strong recent contributions to *Genome Biology* as reviewers. The first interview is with Hyongbum (Henry) Kim, an Associate Professor at Yonsei University College of Medicine in South Korea.

## Please tell us about your research interests

Since 2010, when I become an independent researcher, the primary focus of our lab has been to develop efficient and robust methods that enable genome engineering in various cell types and organisms, including mammalian cells and mice. The ultimate goal of our research is to contribute to the development of novel therapeutic modalities for various diseases using genome engineering methods.

Back in 2010, zinc finger nucleases were the only type of programmable nuclease available for genome editing. The efficiency of generating knockout cells was often low, necessitating significant effort and time for making knockout cell lines. To overcome this problem, Jin-Soo Kim’s group at Seoul National University and our group developed and tested a reporter for zinc finger nuclease activity [1]. Using this reporter we were able to enrich for cells in which the target gene was disrupted by up to 92-fold. We next improved this reporter [2] and applied it to gene editing mediated by the CRISPR-Cas9 nuclease and again achieved enrichment using this system (often more than 10-fold) [3].

Our group also showed the possibility of “safer” genome editing using cell-penetrating peptides to achieve plasmid-free delivery of Cas9 protein and guide RNA. This approach reduces off-target effects and avoids mutagenesis caused by insertion of delivery vectors [4]. Based on these experiences in genome editing, Jin-Soo and I wrote a review article entitled “A guide to genome engineering with programmable nucleases” [5]. Our group also showed, using TALENs, that this gene-disruption approach can convert the blood group of erythroblasts from RhD positive to RhD negative [6].

Currently, we are trying to improve and/or develop genome-wide nuclease libraries for disruption of coding and noncoding elements. Other ongoing projects in the lab include potential therapeutic applications of CRISPR-Cas9 to genetic eye and liver diseases. We are also interested in high-throughput approaches for evaluating the activity of CRISPR-Cas9 based on target sequences. We will continue our research to improve or develop genome editing tools for biomedical research and biotechnology and apply these advanced genome editing methods as therapeutic modalities for various diseases.

## What are your predictions for the field over the next 5 years?

I expect that genome-editing technology will be continuously improved. The efficiency of genome editing will be enhanced and the range of tools will be widened. Currently, CRISPR-Cas9 is the dominant method for genome editing. However, new genome editing tools may emerge, which could be alternative or complementary to CRISPR-Cas9.

I expect that issues associated with off-target nuclease activity may be a less significant problem in the future. We already have unbiased methods for detecting off-target effects and more sensitive methods will be available in the near future. After identifying potential off-target effects, we will be able to predict their potential adverse effects. If a nuclease has off-target effects that could cause significant problems, such as cancer development, then we can choose another nuclease. Furthermore, there are already methods available for reducing off-target effects, such as the use of high fidelity Cas9 nucleases, paired nickases, truncated guide RNAs, or a combination of FokI nuclease with dead Cas9, as well as decreasing the duration of nuclease activity (reviewed in [7]). I would expect that we may not be able to prevent 100 % of the problems induced by off-target effects. However, there will be many cases for which the benefit of genome editing will be much greater than the potential risk of adverse effects caused by off-target activity.

Efficient delivery of genome-editing factors to somatic cells in vivo may remain challenging; a large amount of effort will probably be needed to increase efficiency. Similarly, delivery of RNA interference-inducing factors, such as small interfering RNA (siRNA) or small hairpin RNA (shRNA), is one of the biggest road blocks for the application of this technology. However, transient delivery of genome-editing factors is sufficient for genome editing, in contrast to siRNA or shRNA, which often require continuous delivery. Thus, the burden or difficulty of genome-editing factor delivery to achieve a therapeutic or biotechnological goal may be lower than that for RNA interference.

## What motivates you to provide peer review for journals?

First, the peer review process gives me a good feeling that I am serving the scientific community. When I click the “agree” button to accept the review request, I feel like that I am donating something to the community. Second, in some cases, during the review process, I can get the latest scientific information, which can indirectly affect my research. Third, the improvement of the manuscript by my comments gives me positive satisfaction that I have indirectly contributed to the paper. Given that the number of papers that readers can read is limited, it is important to publish papers that are sufficiently novel, logical, and complete. My comments often improve the manuscript significantly and correct errors in the paper, which will eventually give better information to its readers.

## What changes, if any, would you make to the current system of peer review?

First, I would like to suggest an establishment of a manuscript transfer system across different journal groups. This system could reduce the burden on reviewers and accelerate the publication of the paper. There are good internal transfer systems within some big journal groups. However, transfer of manuscripts between different journal groups is currently difficult.

Second, I would suggest publication of reviewers’ comments even if a paper is rejected from a journal, if the authors want to share with others. There are some journals that publish reviewers’ comments when the paper is accepted. To reduce the probability of unfair review, I would suggest online publication of the rejected manuscript together with the comments when authors choose this option after the paper undergoes peer review. These online publications may be made available at preprint servers or at the journal sites themselves if the editors agree.

I think that these two suggestions may also have some adverse effects. However, given that such changes would be expected to make manuscript publication faster and fairer, they would be cost-effective approaches for facilitating the development of science.

## Have you had any memorably good or bad experiences of peer review, as an author or as a reviewer?

I have had one particularly good experience as a peer reviewer. I was requested to review a paper that was a creative and novel work in this field. However, the manuscript had many errors and there were a lot of points that needed to be addressed before publication. I commented on those issues in detail. Several months later, a much improved version of the paper was sent to me and I was happy to see the improvement. I felt like I was involved in the study.

I had a bad experience regarding peer review as an author, as most scientists have. I submitted a presubmission inquiry about my paper to a journal. The editor sent me positive feedback and I decided to submit my paper to that journal. During the submission, I found that I had the option for double-blind review, in which the authors are anonymous to the reviewers. Because I thought that double-blind review would lead to fairer review, I selected that option. Subsequently, my paper was severely and, in some aspects, unfairly criticized by the reviewers. My guess is that the reviewers may have thought that the anonymous authors would not have sufficient experience to discover something important and new in the research field. Although it is not clear whether double-blind review was a part of the reason for the severe criticisms, I believe that fairer and less biased reviews will be performed if double-blind reviews are mandatory rather than optional.

## References

[1] Kim H, Um E, Cho SR, Jung C, Kim H, Kim JS (2011). Surrogate reporters for enrichment of cells with nuclease-induced mutations. Nat Methods.

[2] Kim H, Kim MS, Wee G, Lee CI, Kim H, Kim JS (2013). Magnetic separation and antibiotics selection enable enrichment of cells with ZFN/TALEN-induced mutations. PLoS One.

[3] Ramakrishna S, Cho SW, Kim S, Song M, Gopalappa R, Kim JS (2014). Surrogate reporter-based enrichment of cells containing RNA-guided Cas9 nuclease-induced mutations. Nat Commun.

[4] Ramakrishna S, Kwaku Dad AB, Beloor J, Gopalappa R, Lee SK, Kim H (2014). Gene disruption by cell-penetrating peptide-mediated delivery of Cas9 protein and guide RNA. Genome Res.

[5] Kim H, Kim JS (2014). A guide to genome engineering with programmable nucleases. Nat Rev Genet.

[6] Kim YH, Kim HO, Baek EJ, Kurita R, Cha HJ, Nakamura Y (2015). Rh D blood group conversion using transcription activator-like effector nucleases. Nat Commun.

[7] Tsai SQ, Joung JK (2016). Defining and improving the genome-wide specificities of CRISPR-Cas9 nucleases. Nat Rev Genet.

